# TEMPO-Oxidized Cellulose Beads as Potential pH-Responsive Carriers for Site-Specific Drug Delivery in the Gastrointestinal Tract

**DOI:** 10.3390/molecules26041030

**Published:** 2021-02-15

**Authors:** Fan Xie, Pieter De Wever, Pedro Fardim, Guy Van den Mooter

**Affiliations:** 1 Drug Delivery and Disposition, Department of Pharmaceutical and Pharmacological Sciences, KU Leuven, 3000 Leuven, Belgium; fan.xie@kuleuven.be; 2 Bio & Chemical Systems Technology, Reactor Engineering and Safety, Department of Chemical Engineering, KU Leuven, 3000 Leuven, Belgium; pieter.dewever@kuleuven.be (P.D.W.); pedro.fardim@kuleuven.be (P.F.)

**Keywords:** TEMPO oxidation, cellulose beads, pH-responsive, controlled drug delivery systems, zero-order release, indomethacin, fenofibrate

## Abstract

The development of controlled drug delivery systems based on bio-renewable materials is an emerging strategy. In this work, a controlled drug delivery system based on mesoporous oxidized cellulose beads (OCBs) was successfully developed by a facile and green method. The introduction of the carboxyl groups mediated by the TEMPO(2,2,6,6-tetramethylpiperidine-1-oxyradical)/NaClO/NaClO_2_ system presents the pH-responsive ability to cellulose beads, which can retain the drug in beads at pH = 1.2 and release at pH = 7.0. The release rate can be controlled by simply adjusting the degree of oxidation to achieve drug release at different locations and periods. A higher degree of oxidation corresponds to a faster release rate, which is attributed to a higher degree of re-swelling and higher hydrophilicity of OCBs. The zero-order release kinetics of the model drugs from the OCBs suggested a constant drug release rate, which is conducive to maintaining blood drug concentration, reducing side effects and administration frequency. At the same time, the effects of different model drugs and different drug-loading solvents on the release behavior and the physical state of the drugs loaded in the beads were studied. In summary, the pH-responsive oxidized cellulose beads with good biocompatibility, low cost, and adjustable release rate have shown great potential in the field of controlled drug release.

## 1. Introduction

Controlled drug delivery systems that can precisely release drugs to specific sites in the human gastrointestinal tract (GIT) at a specific rate have unparalleled advantages compared with traditional oral drug delivery systems, for instance, preventing the side effects of the premature or burst drug release, maintaining a constant plasma concentration for a long time, or reducing the required dosage because of efficient delivery [1,2,3]. Among the various controlled-release systems, the pH-responsive delivery system for oral drug release is of particular interest due to the pH gradient in the GIT from acidic in the stomach (pH 1.0–3.0) to neutral or weakly alkaline (pH 6.0–7.5) in the small intestine [4]. In the past few decades, cross-linked ionic polymer hydrogels or carriers with ionizable functionalities have been developed for pH-responsive drug release at different sites in the human body based on their different swelling characteristics or chemical bond cleavage in different environments. A class of polymers that have been commonly used are derivatives of poly (methacrylic acid) [5,6,7].

Recently, as a ubiquitous macromolecular polymer in nature, polysaccharides are attracting growing attention due to their abundance, biocompatibility, and biodegradability [8,9]. Cellulose, with an annual production of 7.5 × 10^10^ tons, is one of the most abundant bio-renewable sources in the world [10]. Due to its low price, nontoxicity, edibility, and eco-friendly property, cellulose has been considered a promising biomedical material in tissue engineering [11], protein enrichment [12], wound dressings [13], and other fields. It has been reported that spherical cellulose beads with high porosity, high specific surface area, and adjustable pore size can be conveniently prepared from regenerated cellulose, and hence can be used as a potential drug carrier [14]. However, research has shown that the strong hydrogen bonding interactions between the cellulose chains during the drying process will cause “hornification” of the cellulose, meaning both the shrinkage and pore closure are irreversible [15]. This will trap drug molecules into the beads, resulting in poor drug release, which will undoubtedly limit the application of cellulose beads in the drug delivery field. However, if the beads can re-swell at a specific site, the ability to trap the drug in the nontarget area will make it an ideal controlled-release system.

Incorporating carboxyl groups on the cellulose beads not only increases the hydrophilicity of the beads but also improves their reswelling capacity due to the pH-dependent electrostatic repulsive forces between carboxylate groups. This is particularly at pH values above the pKa value of the carboxylic acid, which are encountered in the small intestine. There are several approaches to functionalize carboxyl groups on cellulose, but in recent years, the TEMPO-mediated cellulose oxidation has gained considerable interest as it selectively converts the C6 primary hydroxyl groups of cellulose into carboxyl groups in aqueous conditions [16,17].

In this work, we report a pH-responsive and rate-controllable drug delivery system based on mesoporous oxidized cellulose beads. We studied the effect of the primary oxidant content on the introduction of anions and simultaneously achieved a controlled drug release rate by changing the degree of oxidation. Two model drugs, indomethacin (IND) and fenofibrate (FNB), were used as model drugs to investigate the release kinetics from different oxidized cellulose beads in simulated gastric fluid (pH = 1.2) and phosphate buffer (pH = 7.0). The release curves were fitted to both zero- and first-order kinetic models.

## 2. Results and Discussion

### 2.1. Total Carboxylate Content

**Figure 1:**
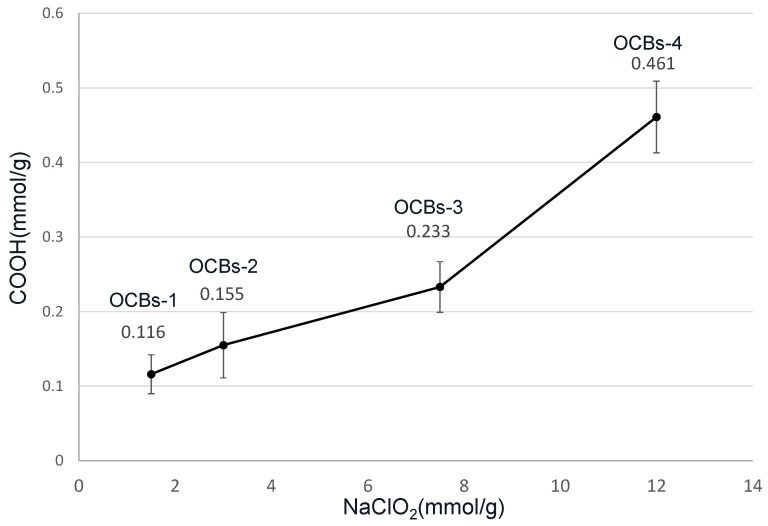
Effect of varying molar ratio of NaClO_2_ on –COOH content.

The results of the carboxylate content analysis in four oxidized cellulose beads measured by conductometric titration are summarized in Figure 1. The results show that carboxylic groups were present in all the samples after 5 h oxidation at 60 °C. An increasing trend can be observed from 0.11 (oxidized cellulose beads (OCBs)-1), 0.15 (OCBs-2), and 0.23 (OCBs-3) up to 0.46 (OCBs-4) mmol/g. The increase in sodium chlorate, which is considered a primary oxidant, clearly increases the degree of oxidation [18].

### 2.2. Morphological Analysis

**Figure 2:**
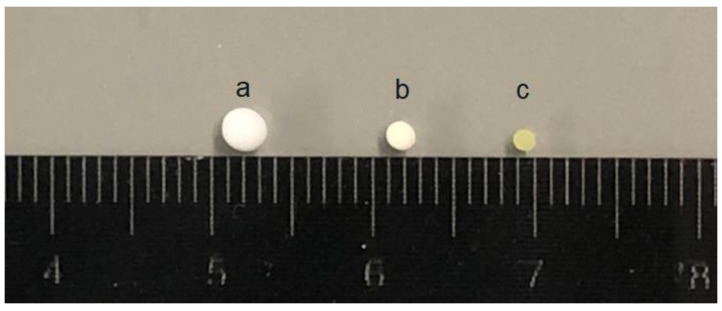
From left to right: (**a**) Freeze-dried oxidized cellulose beads (OCBs)-4 without drug loading. (**b**) OCBs-4 loaded with IND in DCM. (**c**) OCBs-4 loaded with IND in acetone.

**Figure 3:**
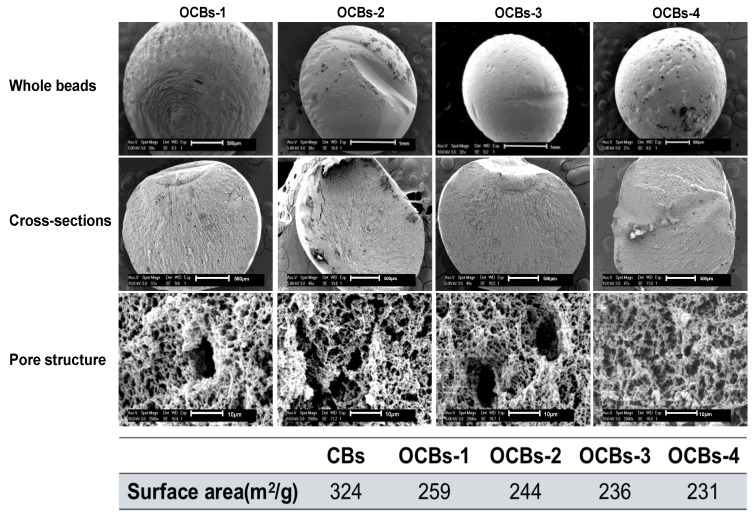
SEM images and surface area of OCBs-1 to OCBs-4. Each row from top to bottom is the morphology of whole beads, cross-section, and porous structure inside, with magnifications 50×, 50×, and 2500×, respectively.

Taking OCBs-4 as a representative, the size of beads before and after drug loading is shown in Figure 2. The diameter of OCBs-4 before drug loading is approximately 2 mm, which slightly decreased after loading with IND in dichloromethane (DCM) and severely shrank after loading with IND in acetone. The scanning electron microscopy (SEM) images provide morphological information of the four different samples, including the surface, cross-section, and pore structure of the beads. As shown in Figure 3, all the samples are spherically shaped with a diameter of approximately 2 mm. No clear difference can be observed in the appearance and cross-section of the four samples. Zooming in on the cross-section of the samples, a dense porous structure can be found inside of the cellulose beads. With the increasing degree of oxidation, the dense network structure still exists in all samples, which suggests that the increasing degree of oxidation hardly affects the beads’ structure. However, the N_2_ adsorption results of the four samples did show differences.

Freeze-dried aerogel beads typically display a low surface area, but by replacing a fraction of the water with tert-butanol (TBA) before the samples are frozen, substantially higher areas can be observed [19]. Unmodified beads display a surface area of 324 m^2^/g, which is considerably higher compared to the areas obtained for polysaccharides by Borisova and coworkers. The key difference is that in their work, gels are formed in a TBA-water mixture, while here, prefabricated beads are washed with a TBA solution before drying [20]. Compared with the unoxidized cellulose beads (CBs), the specific surface area of the beads reduces from 324 to 231 m^2^/g. Although with the increasing degree of oxidation, the decrease in surface area was not dramatic, we still believe that the influence of the oxidation on the beads’ structure exists. N_2_ adsorption results confirm the presence of a mesoporous structure inside the beads. The average pore diameter of the four OCBs is ca. 20–25 nm, although small pores of a few nanometers and macropores over 100 nm are also observed in the pore size distribution (Figure S1).

### 2.3. Swelling Analysis

**Figure 4:**
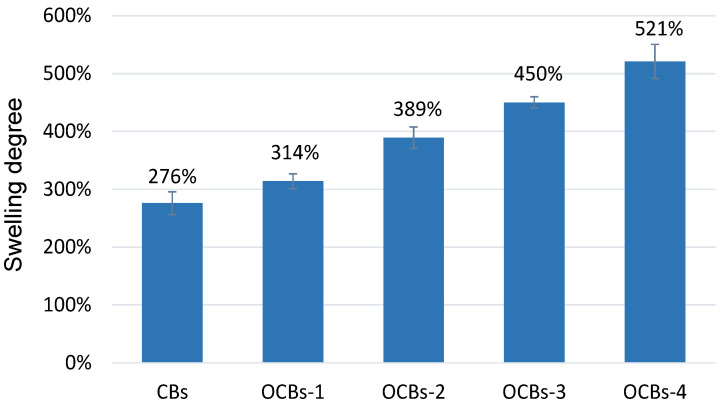
Swelling ratios of cellulose beads (CBs) and four OCBs at pH 7.0 (mean ± SD, *n* = 3).

For controlled drug delivery carriers, the swelling degree is a crucial parameter. The swelling degree of dried porous oxidized cellulose beads is mainly controlled by two important factors, the hydrophilicity of OCBs and the electrostatic repulsion between the carboxylate groups. Swelling studies are carried out at pH 7.0, which is higher than the pKa of –COOH (≈4.0), so the carboxylic acid groups in OCBs are highly deprotonated. As the oxidation degree rises, the stronger electrostatic repulsion between cellulose augments water diffusion into the beads and raises the swelling degree of the beads (Figure 4). After 24 h, the swelling reached equilibrium. Compared with the unoxidized cellulose beads, the swelling degree of OCBs increased from 276 to 521%.

### 2.4. Modulated Differential Scanning Calorimetry (mDSC) and X-ray Powder Diffraction (XRPD) Analysis of IND and FNB-Loaded OCBs

**Figure 5:**
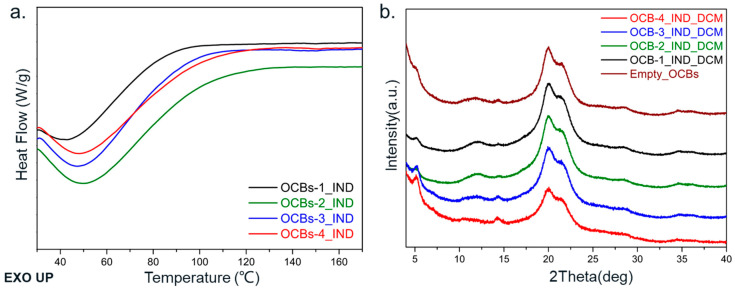
(**a**) DSC thermograms and (**b**) XRPD diffractograms of IND-loaded in OCBs-1 to 4. The baseline curvature in DSC thermograms originates from the evaporation of physically adsorbed water from the cellulose surface.

The results of mDSC and XRPD showed the physical state of the IND and FNB in the cellulose beads. The broad endothermic peak, which appears in the DSC results of IND and FNB, is due to the loss of adsorbed water. In the DSC results of the IND-loaded beads (Figure 5a), no melting peak of IND (stable gamma form: ~162 °C; metastable alfa form: ~154 °C) was observed in any of the samples. The absence of the melting peak reflects the noncrystalline state of the IND loaded in the cellulose pores. The same conclusion can be obtained by XRPD results (Figure 5b). The XRPD diffractograms of all samples exhibit the same diffraction peaks at 2*θ* = 14.7°, 20.1°, and 21.6° as the empty OCBs, which belong to the crystal form of regenerated cellulose II [21]. No Bragg peaks from the crystalline IND were observed, and all the beads maintained the cellulose II crystal form, suggesting that the oxidation reaction did not affect the crystal form of the cellulose beads. The amorphous organization of indomethacin in the beads benefited from the narrow pores of the cellulose beads, which can suppress the crystallization of drugs. The fact that the mesoporous structure (2–50 nm) can limit the crystallization of drugs via geometric constraints has been proven previously [22,23]. Although compared to ordered mesoporous silica, the pore structure of cellulose beads is disordered and has a relatively broad pore size distribution, the existing porous structure of cellulose beads still has a certain restrictive effect on the crystallization of drug molecules.

**Figure 6:**
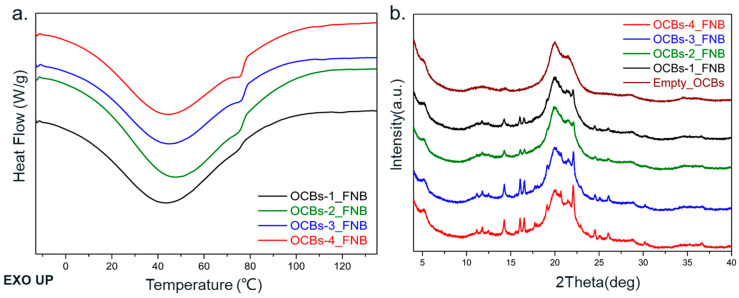
(**a**) DSC thermograms and (**b**) XRPD diffractograms of FNB-loaded OCBs-1 to 4. The baseline curvature in DSC thermograms originates from the evaporation of physically adsorbed water.

Different results were obtained in the case of FNB. The DSC thermograms of FNB show a melting peak around 75 °C, which is characteristic for polymorph II of FNB [24]. As the degree of oxidation increases, the area of the melting peak displays a growing trend, which indicates the increase in crystallinity (Figure 6a). At the same time, in the XRPD diffractograms (Figure 6b), the same increasing trend is present, except for the diffraction peaks belonging to cellulose II, where sharp Bragg diffraction peaks belonging to crystalline FNB are also observed. With the increase in oxidation degree, OCBs-3 and OCBs-4 show diffraction peaks of FNB with higher intensity than OCBs-1 and 2. The difference between FNB and IND may be related to the difference in physical stability. FNB is known for its low glass transition temperature. The glass transition temperature of −20 °C is much lower than that of indomethacin, which makes it more likely to crystallize when the beads are dried and stored at room temperature [25].

### 2.5. In Vitro Release Results

#### 2.5.1. IND-Loaded OCBs

**Figure 7:**
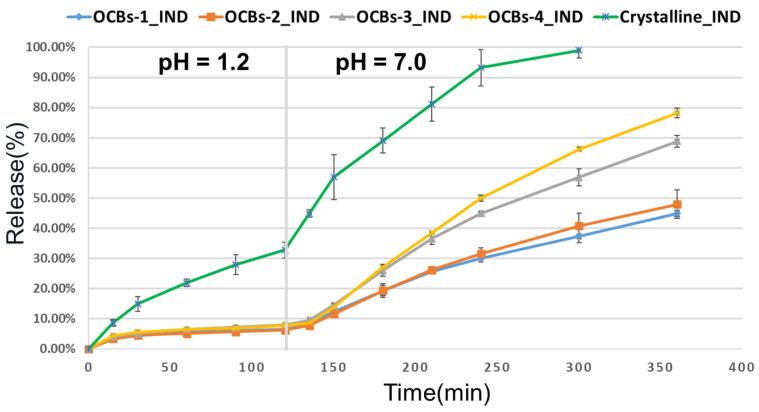
Release profiles of IND from OCBs in different pHs. The depicted results are mean ± SD, *n* = 3.

The release profile of OCBs-1 to 4 loaded with IND solution in acetone is shown in Figure 7. The drug release of all samples is less than 10% at pH 1.2 for the first two hours, which proved the good drug retention effect of the beads. The results at pH 7.0 for the subsequent four hours show that the release rates varied with the degree of oxidation of the beads: The higher the oxidation degree, the faster the release rate. After four hours, approximately 80% of the IND could be released from the OCBs-4 beads, which is twice the amount in the case of OCBs-1. It is worth mentioning that as IND is a weakly acidic drug, the pure crystalline IND shows a fast dissolution rate at pH 7. This premature release is disadvantageous for targeted delivery in the lower intestine. However, in our work, even the case of OCBs-4, which has the fastest release rate, still retained 20% of the drug inside of the beads after six hours and maintained a sustained release trend. Therefore, it is possible to design different systems for drug release at different positions of the intestine by adjusting the degree of oxidation of the cellulose beads.

**Figure 8:**
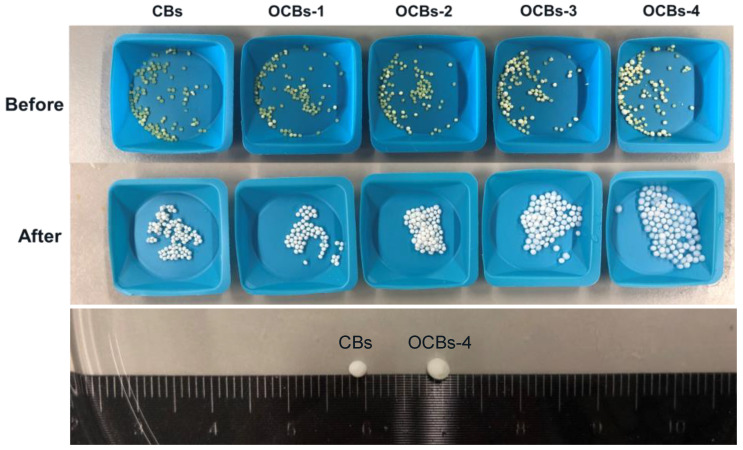
(Top) The appearance of IND-loaded CBs and OCBs before and after drug release. (Bottom) Different swelling degrees of CBs and OCBs-4 after drug release at pH = 7.0.

The appearance of the beads before and after the drug release test is shown in Figure 8. All the beads shrank severely due to the capillary forces acting on the pore walls after drug loading, and kept shrinking at pH 1.2, thus retaining the drug inside. Compared with the unoxidized beads, OCBs-3 and 4 showed significant swelling during dissolution due to the stronger electrostatic repulsion between –COO^−^. At the same time, the carboxylic acid of IND would be ionized into carboxylate at pH 7. Thus, the release of IND from OCBs at pH 7.0 is the combined effect of beads re-swelling and the electrostatic repulsion between –COO^−^ on beads and IND.

**Figure 9:**
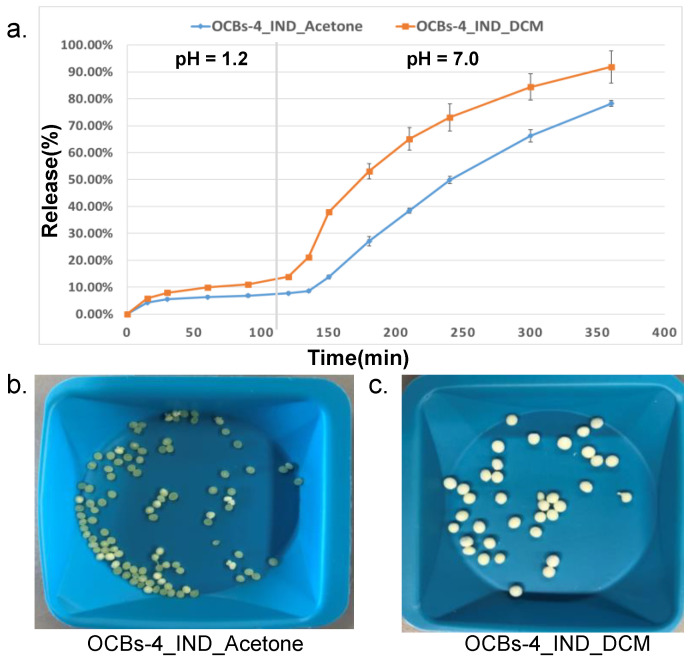
(**a**) Release profiles of OCBs-4_IND loaded in DCM and acetone. The results are presented as mean ± SD, *n* = 3. (**b**) Appearance of OCBs-4 after loading with IND in acetone and (**c**) appearance of OCBs-4 after loading with IND in DCM.

At the same time, we also considered the impact of drug-loading solvents on beads’ morphology and drug release behavior. Dichloromethane (DCM), which has a faster evaporation rate than acetone at room temperature, was applied to investigate the influence of the solvent on drug loading and drug release. Apparently, the beads loaded with IND in DCM exhibited (Figure 9c) a lower shrinkage than in acetone (Figure 9b) and nearly maintained the original size of the beads. As shown in Figure 9a, a burst release was observed in both samples in the first thirty minutes at pH 1.2, and then the release rate remained constant, which suggested that part of the drug was on the surface of the beads rather than completely included in the pores. At pH 7.0, OCBs-4_IND_DCM showed a higher extent of release and higher release rate, especially in the early stage of release. This is due to the lower degree of shrinkage, which allows a higher amount of accessible water and creates more open pores.

#### 2.5.2. FNB-Loaded OCBs

**Figure 10:**
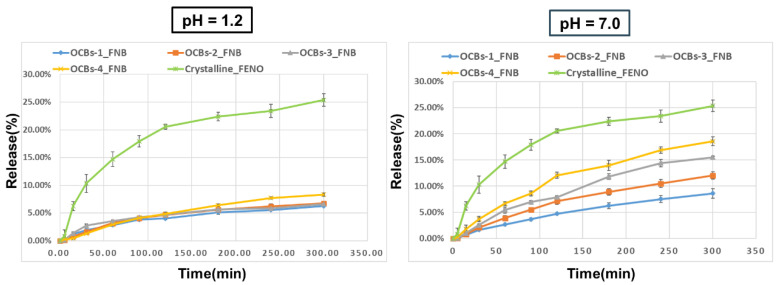
Release profiles of FNB from OCBs at pH = 1.2 (left) and 7.0 (right). The results are presented as mean ± SD, *n* = 3.

In addition, we also evaluated the capacity of OCBs for drugs with different chemical properties on drug loading and controlled release. Different from the high solubility of indomethacin at pH 7.0, fenofibrate is a neutral drug that exhibits extremely low solubility at any pH value. Similarly, the acetone solution of FNB was used for drug loading, and the release experiment was carried out for 5 h at pH 1.2 and 7.0. The release profiles (Figure 10) show that all samples released extremely low amounts at pH 1.2 (OCBs-1–3 was 6% and OCBs-4 was 8% after 5 h). The release of all samples at pH 7.0 was higher than the release at pH 1.2, especially for OCBs-4, where the release doubled (from 8.61 to 18.58%). Similar to IND, with increasing oxidation degree, the release rate and release amount increased. The final release of FNB was much lower than that of IND, which can be explained by the low solubility of FNB at pH = 7.0 and the lack of electrostatic repulsion between FNB and OCBs. Although the final amount of FNB released from the OCB beads was still lower than that of crystalline FNB, we observed the pH responsiveness of the OCBs and the ability to adjust the drug release rate through different oxidation degrees.

### 2.6. Kinetics of the Drug Release

The release data of four OCBs at pH 7.0 were fitted to the zero-order and first-order kinetic models, and the different release rate constants (K_0_ and K_1_) values with their corresponding determination coefficient R^2^ values are presented in Table 1. The release of IND loaded from IND/acetone solution shows high determination coefficients with both zero-order and first-order models. According to the R^2^, OCBs-1 to 3 are better fitted by a first-order release model, while OCBs-4 is closer to a zero-order release, indicating that OCBs-4 achieves a nearly constant release rate of IND. It is worth noting that the release profiles of the beads loaded by IND/DCM displays a difference. The R^2^ value of the first-order model (R^2^ = 0.9788) is much higher than in the case of the zero-order model (R^2^ = 0.8686). Meanwhile, from the visual observation, the curve of OCBs-4_IND_DCM is more in line with the “fast then slow” first-order release, and the larger K_1_ indicates a faster release rate than OCBs-4_IND_Acetone. Therefore, the release kinetics of OCBs loaded with IND/acetone is more conducive to maintaining a constant blood concentration and is more conducive to achieving targeted delivery and controlled release of the lower intestinal tract. For the beads loaded with FNB, the R^2^ values of zero-order equations for all samples are higher than that of the first-order equation. Although the difference is minor, these results support that the release of FNB from four OCBs is closer to a zero-order release.

The release data of FNB at pH 1.2 are also fitted with the zero-order and first-order equations to compare the effects of different pHs on the release behavior. As shown in Table 2, all samples showed absolutely higher determination coefficients with the zero-order model than with the first-order, which was different from the release at pH 7.0. The closer K_0_ value suggests the almost identical release rate of four OCBs. It is worth mentioning that due to the extremely low release of FNB at pH = 1.2, the fittings with both zero-order and first-order equations were poor.

**Figure 11:**
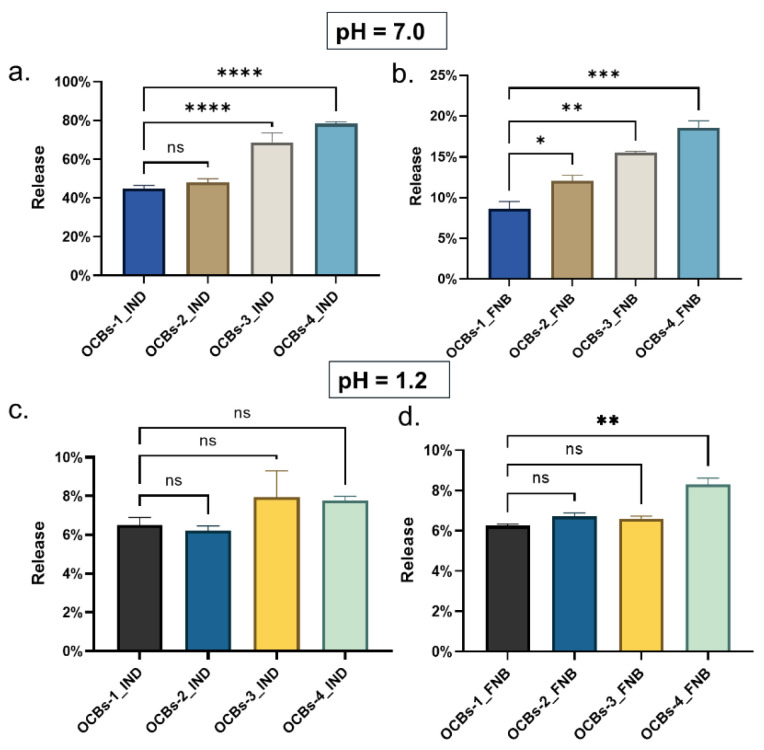
Comparison of the final release percentage of IND (**a**) at pH = 7.0 and (**c**) 1.2, and FNB (**b**) at pH = 7.0 and (**d**) 1.2 from four oxidized beads. ns: not significant, * *p < 0.05*, ** *p* < 0.01, *** *p* < 0.001, and **** *p* < 0.0001.

According to the results of ANOVA (Figure 11), the final IND release for OCBs-1 and OCBs-2 at pH 7.0 is not significantly different (Figure 11a). As the oxidation degree of these beads is very similar (see Figure 1), the effect on the release rate is negligible, but as the degree of oxidation increases, a significant difference is noted. Significant differences between all samples are observed in the case of FNB released at pH = 7.0 (Figure 11b). As the degree of oxidation increases, the differences increase similarly. By contrast, except between OCBs-1_FNB and OCBs-4_FNB, the final release between other samples does not show a significant difference (Figure 11d), which proves that all beads have effective retention of drugs at pH = 1.2.

## 3. Materials and Methods

### 3.1. Materials

Dissolving pulp from birch wood, obtained from Stora Enso (Helsinki, Finland), was used as a cellulose source and pretreated as described earlier [26]. Sodium hydroxide (97%) was purchased from MilliporeSigma™ (Darmstadt, Germany). Glacial acetic acid (99%) and NaCl (99.8%) were purchased from CHEM-LAB. HCl was purchased from Fisher Scientific U.K. Limited (Loughborough, UK), and fenofibrate was purchased from Hangzhou Apichem Technology CO LTD (Hangzhou, China). TEMPO (98%), indomethacin, HNO_3_ (37%), and tert-butanol (99%) were obtained from ThermoFisher (Kandel, Germany). Sodium lauryl sulfate (SLS) was obtained from Merck KGaA (Darmstadt, Germany). Urea (99.5%), sodium acetate acid (99%+), NaClO (10–15%), NaClO_2_ (80%), acetone (99%), methyl sulfoxide (DMSO, 99.9%), acetonitrile (ACN, 99%), and dichloromethane (DCM, 99.9%) were purchased from ACROS Belgium (Geel, Belgium).

### 3.2. Oxidation of Mesoporous Cellulose Beads

Cellulose beads were prepared according to Trygg et al. [15]. Briefly, 5% pretreated cellulose pulp was dissolved in 7% NaOH/12% urea aqueous solution. The mixture was subsequently cooled to −13 °C while stirring for 2 h until the cellulose pulp was completely dissolved and visually transparent. A syringe pump was used to add the cellulose dropwise into a 2 M nitric acid solution at 25 °C to induce coagulation. After 2 h, the cellulose beads were washed with an excess of deionized water for 30 min and stored in deionized water before the oxidation reaction was initiated.

In a 200 mL flask, 13 g nondried cellulose beads (equivalent to 1 g dry cellulose, with 92% water) were immersed in 100 mL phosphate buffer (50 mM; pH 4.6) and kept overnight. Subsequently, TEMPO/NaClO_2_/NaClO was added in different ratios. As the primary oxidant, the proportion of NaClO_2_ is critical to the degree of oxidation. Therefore, different amounts of NaClO_2_ were added while keeping the molar ratio of the other two reagents constant (Table 3) to synthesize four different types of oxidized beads, OCBs-1 to OCBs-4. The reaction was carried out under continuous stirring (100 rpm) at 60 °C for 5 h. The chlorine gas generated during the reaction was collected with a saturated NaOH solution. Excess ethanol was added to quench the reaction and the beads were washed and stored in deionized water. To avoid the swelling of the beads during the washing process, which may damage the pore structure, the pH of deionized water was adjusted to 3.5.

### 3.3. Determination of Carboxylate Content

The carboxylate content of oxidized cellulose beads was determined by conductometric titration based on the procedure reported by Paula et al. [27]. In short, 13 g wet OCBs-1 to 4 (approx. 1 g dry cellulose) were immersed into 0.1 mol/L HCl solution for 15 min to protonate the beads, and subsequently washed repeatedly with deionized water to remove excess acid until the conductivity of the washed filtrate was less than 5 μS/cm. The beads were crushed and suspended into a mixture of 490 mL of distilled water and 10 mL of 0.05 mol/L NaCl solution. The suspension was titrated with 0.05 mol/L NaOH. The total carboxylate content was calculated according to Equation (1):

(1) $$C_{\mathrm{COOH}}=\frac{C\left(V-V1\right)}{m}$$

where

C_COOH_ is the total acidic group content, in μmol/g;

C is the concentration of the NaOH solution, in μmol/L;

V is the volume of the NaOH consumed in the whole process, and V1 is the consumption of protons neutralization liberated by NaCl, in liters;

m is the weight of dry sample, in grams.

### 3.4. Freeze-Drying

To maintain the pore structure and surface area of porous cellulose beads, an optimized freeze-drying process was used to prepare dry beads. Specifically, a tert-butanol (TBA)/water cosolvent system was used due to the smaller surface tension of TBA and the “gentle” solidification of 90% TBA/10% water eutectic crystals formed during freeze drying [20]. Using a solvent exchange process, the beads were immersed for one hour into consecutive TBA/water mixtures with increasing TBA content ranging from 22.5%, 45%, and 67.5% to 90%. After the solvent exchange procedure, the beads were flash-frozen using liquid nitrogen and transferred to an ALPHA 1–4 LSC, CHRIST freeze-dryer (Martin Christ Gefriertrocknungsanlagen GmbH). The following operational conditions were maintained for three consecutive days: Ice condenser set at −85 °C and pressure of 0.500 mbar.

### 3.5. Characterization of Beads

#### 3.5.1. Scanning Electron Microscopy (SEM)

The surface and internal morphology of the freeze-dried beads were characterized by SEM. To prepare the samples, both intact beads and cross sections were attached to carbon tape and coated with platinum (SCD-030 Balzers Union sputter-coater, Balzers, Liechtenstein). The particles were then visualized with a Phillips XL30 SEM-FEG (Philips, Eindhoven, The Netherlands) including a Schottky emission electron gun. A beam of 20 kV and a conventional Everhart–Thornley-type detector was used.

#### 3.5.2. Nitrogen Physisorption and Calculation

The specific surface area of all freeze-dried cellulose beads was measured by a 3P meso 222 sorption analyzer (3P INSTRUMENTS, Odelzhausen, Germany) with N_2_ as the sorption gas at −196 °C. All samples were first degassed at 60 °C for 6 h under nitrogen flushing before analysis. The specific surface area was calculated by the Brunauer–Emmett–Teller (BET) method.

#### 3.5.3. Swelling Analysis

The swelling degree of freeze-dried cellulose beads with different degrees of oxidation was determined by monitoring the weight gain of beads in phosphate buffer (pH = 7.0), using nonoxidized cellulose beads (CBs) as a reference. The freeze-dried beads were weighted (denoted as Wdry) and then immersed in a phosphate buffer solution at room temperature for 24 h. After removal of the excess water on the surface, the beads were weighed (denoted as Wwet) and the swelling degree (SD) was calculated according to Equation (2). Each sample was tested in triplicate.

(2) $$\mathrm{SD}=\frac{W_{\mathrm{wet}}-W_{\mathrm{dry}}}{W_{\mathrm{dry}}}100\left(\%\right)$$

### 3.6. Drug Loading

The incipient wetness procedure was used to load ca. 20% of model drugs into oxidized cellulose beads [28]: 1 mL of a 20 mg/mL drug solution was slowly added to 100 mg of cellulose beads. To investigate the effect of solvent on drug loading and release, IND was dissolved in DCM and acetone, while in the case of FNB, only acetone was used. The loaded beads were dried at 25 °C in a vacuum oven overnight.

The drug load was determined by the difference in weight of the cellulose beads before and after loading and confirmed by assay. Immersing the beads into DMSO overnight after the dissolution test and measuring the content of drugs by HPLC, the total amount of drug was the sum of the released drug during dissolution and the drugs remaining in the beads.

### 3.7. Solid-State Characterization

#### 3.7.1. Modulated Differential Scanning Calorimetry (mDSC)

The physical state of model drugs in beads was investigated with mDSC using a Q2000 (TA Instruments, Leatherhead, UK) with a heating rate of 2 °C/min and purged with dry nitrogen at a flow rate of 50 mL/min. Approximately 10 mg beads (≈2 mg drugs) were accurately weighed into large aluminum DSC pans (TA Instruments, Zellik, Belgium), and the beads were gently pressed with lids to make better thermal contact with the DSC pans. The heating range in the case of IND was from 25 to 180 °C and from −20 to 135 °C in the case of FNB. Universal Analysis 2000 software (TA Instruments, New Castle, DE, USA) was used for data processing.

#### 3.7.2. X-ray Powder Diffraction (XRPD)

XRPD experiments were carried out using an automated X’Pert PRO diffractometer (PANalytical, Almelo, The Netherlands) with a Cu tube (Kα λ = 1.5418 Å) and a generator set at 45 kV and 40 mA. Measurements in transmission mode were performed at room temperature (RT), using Kapton Polyimide thin films (PANalytical, Westborough, MA, USA). A continuous scan mode from 4 to 40° 2θ with a 0.0167° step size and 400 s counting time was applied. X’Pert Data Viewer (Version 1.7, PANalytical, Almelo, The Netherlands) was used to analyze the obtained diffractograms.

### 3.8. In Vitro Drug Release Testing

The release behavior of the model drugs from OCBs-1 to OCBs-4 beads was investigated at 37 °C under nonsink conditions using a SR8PLUS dissolution station (SpectraLab Scientific Inc., Markham, ON, Canada) with paddle apparatus II, and a rotation speed of 50 rpm. Each sample was tested in triplicate. More specifically, the release from beads loaded with 20% of IND was first performed in 150 mL simulated gastric fluid (SGF) containing 0.5% sodium lauryl sulfate (SLS, pH 1.2) for 2 h, and subsequently in 150 mL phosphate buffer (PBS, pH 7.0) for another 4 h. Then, 3 mL samples were taken at different time points, filtered through a CHROMAFIL^®^ O-45/15 MS PTFE filter (pore size 0.45 μm, MACHERY-NAGEL GmbH & Co. KG, Düren, Germany), and quickly replaced with the same volume of fresh medium. The filtered samples were used to determine the concentration of IND by HPLC. For beads loaded with 20% of FNB, the drug release tests were carried out separately in SGF with 0.25% SLS (pH 1.2) and PBS with 0.25% SLS (pH 7.0) for 5 h. Samples were taken at different time points and filtered with the same filter.

HPLC-analysis of IND was performed using a Merk-Hitachi LaCrom system equipped with a Nucleodur C18 column (150 mm, 4.6 mm ID, particle size 5 µm) obtained from Macherey-Nagel (Düren, Germany). For all samples, the flow rate of the mobile phase was 1 mL/min and the injection volume of samples was 20 µL. The mobile phase for analysis of IND consisted of 20 mM sodium acetate buffer (pH 3.50)-ACN (40–60%; *v/v*), resulting in a retention time of 5.5 min. For FNB, the mobile phase consisted of 0.85% phosphoric acid (pH 1.50)-ACN (10–90%; *v/v*), resulting in a retention time of 5 min.

### 3.9. Drug Release Kinetics 

The release kinetics of model drugs from the OCBs was studied by fitting the release curve to zero-order Equation (3) and first-order Equation (4) kinetic equations [29],

(3) $$M_{t}/M_{\infty }={\;K}_{0}t$$

(4) $$\ln(1-\frac{M_{t}}{{\;M}_{\infty }})=-K_{1}t,$$

where M_t_/M_∞_ is the fraction of drug release at time t, and K_0_ and K_1_ are the kinetic constants of the two equations, respectively.

### 3.10. Statistical Analysis of Drug Release Testing

All release experiments were performed in triplicate, and results were expressed as mean ± standard deviation (SD) and indicated as error bars in the release profiles. The final release data of IND and FNB at pH 1.2 and 7.0 were statistically analyzed using one-way analysis of variance (ANOVA) and Tukey’s multiple comparisons test. *p* values < 0.05 were considered statistically significant. Statistical analysis was carried out using Graph Pad Prism 9 software (Graph Pad Software, San Diego, CA, USA).

## 4. Conclusions

Cellulose beads were prepared and anionic groups were introduced by the TEMPO/NaClO/NaClO_2_-mediated oxidation process. Four OCBs with different oxidation degrees were successfully obtained by adjusting the amount of NaClO, resulting in an increased carboxylate content in the beads. The morphological characterization of the four OCBs confirmed the existence of a dense porous structure, with a specific surface area above 200 m^2^/g. The specific surface area decreased with the degree of oxidation. The swelling capacity of OCBs increased with the degree of oxidation due to the higher hydrophilicity of the beads’ surface. The larger quantities of carboxylic acid groups enhanced electrostatic repulsion between the different surfaces.

The drug release experiments of the two model drugs proved that OCBs had a pH-responsive release ability, i.e., almost no release at pH 1.2, but a controlled release rate at pH 7. As the degree of oxidation increased, the release rate became faster. The statistical analysis showed that there were significant differences in the release properties of beads with different oxidation degrees. Due to the difference in chemical properties, the release of IND was higher than that of FNB. It was found from the kinetics studies that the release behavior of OCBs loaded with IND/acetone solution and FNB/acetone solution was closer to a zero-order process, indicating a constant release rate. However, OCBs loaded with IND/DCM solution exhibited a first-order profile, which suggests a rapid release rate at the early stage followed by a continuously slowing down of the rate. In summary, the drug release from pH-responsive OCBs was affected by the degree of oxidation, the chemical properties of the model drugs, and the drug-loading solvent.

## Figures and Tables

**Table 1:** Results of fitting the release data of IND and FNB at pH 7.0 with zero-order and first-order kinetic models.

Samples	Zero-Order		First-Order	
	k_0_	R^2^	k_1_	R^2^
OCBs-1_IND_Ace	0.0957	0.9695	0.1264	0.9904
OCBs-2_IND_Ace	0.1076	0.9736	0.1395	0.9932
OCBs-3_IND_Ace	0.1583	0.9731	0.2235	0.9862
OCBs-4_IND_Ace	0.1899	0.9769	0.2704	0.9680
OCBs-4_IND_DCM	0.1755	0.8686	0.4422	0.9788
OCBs-1_FNB_Ace	0.0163	0.9739	0.0200	0.9435
OCBs-2_FNB_Ace	0.0230	0.9536	0.0280	0.9292
OCBs-3_FNB_Ace	0.0306	0.9609	0.0380	0.9490
OCBs-4_FNB_Ace	0.0348	0.9411	0.0477	0.9215

**Table 2:** Results of fitting the release data of FNB at pH = 1.2 with zero-order and first-order kinetic models.

Samples	Zero-Order		First-Order	
	k_0_	R^2^	k_1_	R^2^
OCBs-1_FNB_Ace	0.0114	0.8846	0.0150	0.7501
OCBs-2_FNB_Ace	0.0130	0.8697	0.0171	0.7911
OCBs-3_FNB_Ace	0.0114	0.8133	0.0179	0.5807
OCBs-4_FNB_Ace	0.0174	0.9439	0.0203	0.9352

**Table 3:** Dosage of reagents in four different oxidation samples (mmol/g).

	OCBs-1	OCBs-2	OCBs-3	OCBs-4
**NaClO_2_**	1.50	3.00	7.50	12.0
**TEMPO**	0.200			
**NaClO**	0.600			

## Data Availability

The data presented in this study are available on the request from the corresponding author.

## Supplementary Materials

The following are available online: Supplementary Materials S1: Pore distribution of (A) OCBs-1, (B) OCBs-2, (C) OCBs-3, and (D) OCBs-4.
