# Supplementary figures and images for: TEMPO-Oxidized Cellulose Beads as Potential pH-Responsive Carriers for Site-Specific Drug Delivery in the Gastrointestinal Tract
Source: Molecules. 2021 Feb 15;26(4):1030. doi: 10.3390/molecules26041030 (PMC7919685; doi:10.3390/molecules26041030)

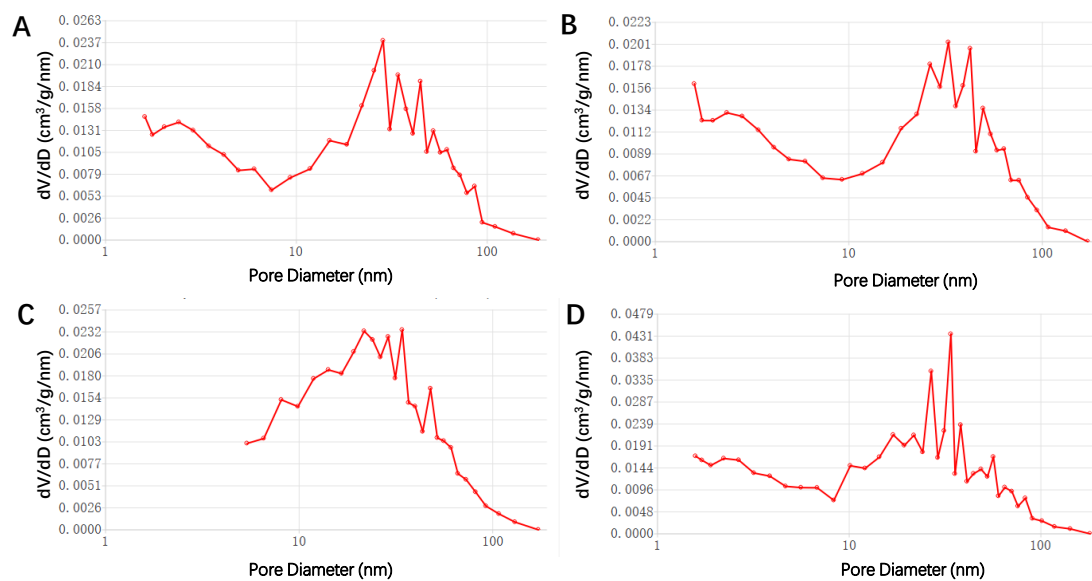

Figure S1. Pore distribution of (A) OCBs-1, (B) OCBs-2, (C)OCBs-3 and (D)OCBs-4.

Supplement: Supplementary file 1 [file molecules-26-01030-s001.pdf]
